# Breakdown of the compositional data approach in psychometric Likert scale big data analysis: about the loss of statistical power of two-sample t-tests applied to heavy-tailed big data

**DOI:** 10.1186/s40708-025-00253-2

**Published:** 2025-04-07

**Authors:** René Lehmann, Bodo Vogt

**Affiliations:** 1 Chair in Business Administration, esp. in Empirical Economics, and Health Economics, Otto von Guericke University, Magdeburg, Germany; 2 Ifes Institute of Empiricism and Statistics, FOM University of Applied Science, Essen, Germany

## Abstract

Bipolar psychometric scale data play a crucial role in psychological healthcare and health economics, such as in psychotherapeutic profiling and setting standards. Creating an accurate psychological profile not only benefits the patient but also saves time and costs. The quality of psychotherapeutic measures directly impacts grant funding decisions, influencing managerial choices. Moreover, the accuracy of consumer data analyses affects costs, profits, and the long-term sustainability of decisions. Considering psychometric bipolar scale data as compositional data can enhance the statistical power of well-known paired and unpaired two-sample t-tests, supporting managerial decision-making and the development or implementation of health interventions. This increase in statistical power is observed when the central limit theorem (CLT) holds true in statistics. Through stochastic simulation, this study explores the impact of violating the CLT on statistical power of the unpaired t-test under heavy-tailed data generating processes (DGPs) with finite variance. The findings reveal a reduction in statistical power based on specific parameters like the psychometric limit of quantification, the number of items in a questionnaire, the response scale used, and the dispersion of the DGP.

## Introduction

Adequate psychometric profiling contributes to various scientific areas including health psychology (e.g., measurement and assessment of personality, prediction of human behaviour), neurology (e.g., cognition and pre-post-comparisons), health economics (e.g., efficacy of treatments) and econometrics (customer satisfaction and attitudes). Psychological big data is used for validating predictive models of human behaviour and attitudes by applying a model developed on one dataset to a separate set of data or hold-out sample [1]. Concerning health psychology, the statistical analysis of individual psychometric data and big data sets contributes to the derivation of standards and the evaluation of the success of psychotherapeutic measures, e.g., via individual psychometric profiling and machine learning algorithms [2, 3]. Psychotherapeutic treatment and behaviour prediction both depend on the correct specification of personality facets and attitudes of individuals. Correct unbiased psychometric profiling can support the selection of apposite healthcare measures, reduce the costs of a treatment and save time. Moreover, the increase of patient welfare contributes to medical ethics [4].

Effective psychometric profiling plays a crucial role in various scientific fields, including healthcare, health economics, and the prediction of human behavior. Psychological big data is utilized to validate predictive models of human behavior and attitudes by applying a model developed on one dataset to a separate set of data or hold-out sample [1]. In the realm of health psychology, the statistical analysis of individual psychometric data and large datasets aids in establishing standards and evaluating the effectiveness of psychotherapeutic interventions. This can be achieved through individual psychometric profiling and the utilization of machine learning algorithms [2, 3].

The success of psychotherapeutic treatment and behavior prediction hinges on accurately capturing the personality traits and attitudes of individuals. Unbiased psychometric profiling supports the selection of appropriate healthcare interventions, leading to cost savings and time efficiency. Furthermore, enhancing patient well-being contributes to medical ethics [4].

Biased results, however, can be misleading because effects (e.g., significant improvements and coherences) can be overseen. Moreover, statistical biases can pretend inexistent effects (e.g., biased estimates of effect size) corrupting managerial decision making.

In a recent study, [4] uncovered the compositional structure of data from bipolar scales questionnaires. The study focused on the magnitude of agreement (OMA) and disagreement (OMD) towards statements within the questionnaire. OMA and OMD are interrelated and together form a bivariate compositional data point [5]. Neglecting this complementary information can introduce significant bias into subsequent statistical analyses [6], potentially reducing statistical power. By applying the isometric log-ratio (ilr) transformation, researchers can obtain interval-scaled real data [7–9] and achieve unbiased results [10].

When the central limit theorem of statistics (CLT) is upheld [4], the ilr approach enhances the statistical power of well-known tests such as the paired (PAIRED) and unpaired (UNPAIRED) two-sample t-tests [11, 12], as well as the correlation test based on the t-distribution [4, 13]. The increase in power stems from the unbiasedness of parameter estimates achieved through the ilr approach [4, 11, 13]. Furthermore, research by [14, 15] has shown that the ilr transformation leads to more likely normally distributed means of item response data.

In the context of PAIRED and UNPAIRED tests, questions arise regarding potential violations of the CLT. This article addresses these concerns by simulating the effects of Laplace distributed data generating processes (DGPs) on the statistical power of the PAIRED and UNPAIRED. Additionally, the study examines how factors such as the psychometric limit of quantification (LOQ), the number of items in a questionnaire, the number of responses $K=k+1$ of the response scale (RS), and the total dispersion of the DGP ($s^2$) impact statistical power.

## Materials and methods

This section provides a brief overview of bipolar psychometric scales, compositional data structures and the ilr approach. The ilr and the inverse ilr transformation are introduced as well as the principles of the simulation study.

### Psychometric bipolar scales and the LOQ

**Fig. 1:**
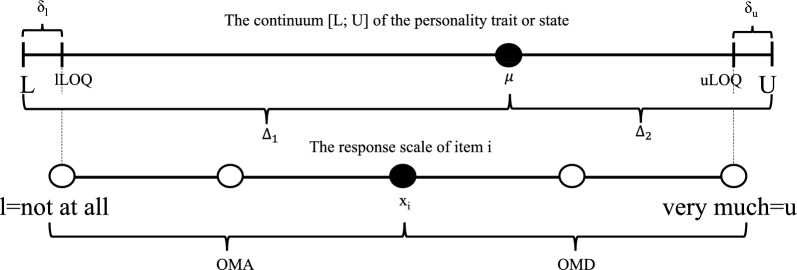
Illustration of the different types of scales used in psychometrics. The continuum [L; U] represents the TS. The lower scale represents the RS. Figure according to [4]

It is necessary to distinguish between the trait scale (TS) of a personality trait (i.e., the continuum of all possible manifestations of a trait) and a Likert scale (LS), i.e., a set of items. While the continuous TS ranges from a minimum value (e.g., $L=0$) to a maximum value (e.g., $U=100$) the LS provides an estimate of a test person’s order of magnitude of the trait (OMT). A RS is a format used to quantify a test person’s OMA. Often, the OMA is represented by a verbal response (e.g., ranging from “not at all” to “very much”) where verbal responses are associated with numerical values (e.g., $1,\ldots ,5$) [16, 17]). In practice, arithmetic means or sums of item response values (i.e., means or sums of OMA values) are used to estimate the OMT [18–20]. An illustration of the different types of scales is presented in Fig. 1

The continuum [L; U] contains all possible individual manifestations of a construct ranging from a minimum value L (e.g., non-openness to anything) to a maximum value U (e.g., openness to everything). A person’s order of magnitude of the construct (say, $\mu$) is located within these bounds. Moreover, the complements $\Delta _1$ and $\Delta _2$, both represent the order of magnitude of the construct. We have $\Delta _1+\Delta _2=U-L$. For example, set L=0, U=100, $\mu =70$, $\Delta _1=70$ and $\Delta _2=30$.

The psychometric scale consists of different items $i=1,\ldots ,I$ associated with a responses scale, e.g., ranging from l=“not at all” to u=“very much”. As the items cannot cover all aspects of the construct the lower (l) and upper (u) limit of the response scale are different from L and U reflecting the lower (lLOQ) and upper (uLOQ) limit of quantification. The edge area of the construct scale which is not covered by the items and their respective response scale are named $\delta _l$ and $\delta _u$.

Any response $x_i$ towards an item assertion reflects the order of magnitude of agreement ($d_1$) and the order of magnitude of disagreement ($d_2$) towards the item assertion. For example, let l=lLOQ=2.5, u=uLOQ=97.5, $x_i=73.75$, $d_1=50$, $d_2=50$, $\delta _l=[0;2.5)$ and $\delta _u=(97.5;100]$). That is, $x_i$ estimates the unknown value of $\mu$ and the pair $(73.75,26.25)^T$ denotes a so-called (bivariate) compositional data point.

Due to imperfect knowledge, uncertainty about situations and a complex environment [21–23] neither a specific item nor a set of items can cover all aspects of a trait. For example, someone who chose the item response “strongly agree” does not agree to the item assertion concerning all possible circumstances because it is impossible for the individual to take into account all facts. Consequently, the RS covers a proportion of the TS and there exists a lower and an upper LOQ (say, lLOQ and uLOQ). The cardinality of the edge areas not covered by the RS (say, $\delta _l$ and $\delta _u$) equals the proportion $(0; 1)\ni p=\left| \delta _l\cup \delta _u \right|$, that is, *p* quantifies the value of the LOQ. For an illustration refer to Fig. 1.

### The compositional structure of bipolar scales data

Concerning the TS, any limit values $L, U\in {\mathbb {R}}$ can be assumed as long as $L<U$ is satisfied, e.g. $L=0$ and $U=100$. For example $\mu _1=0.5$ is the midpoint of the TS $[L; U]=[0; 1]$ whereas $\mu _2=50$ represents the midpoint of the TS $[L; U]=[0; 100]$. $\mu _1$ and $\mu _2$ both represent the same order of magnitude of the trait but on different scales, so *L* and *U* can be chosen arbitrarily.

Without loss of generality consider a RS $r=\{r_1,\ldots ,r_{k+1}\}$ with $r_1=1$, $r_{k+1}=k+1$, $k\in {\mathbb {N}}$, $r_{s+1}-r_s=1$ $\forall s\in {1,\ldots ,k}$ (e.g., the discrete scale $\{1,2,3,4,5\}$ of $k+1=5$ categories ranging from “not at all (1)” to “very much (5)”).

Let $p\in (0;1)$ quantify the LOQ. Symmetric values of lLOQ and uLOQ are assumed, that is, $lLOQ=100\cdot p/2$ and $uLOQ=100(1-p/2)$. Therefore, the edge areas are also symmetric with $\left| \delta _l\right| =\left| \delta _u\right| =p/2$.

Let $x'\in \{r_1,\ldots ,r_{k+1}\}$ be an observed response value and let $p\in (0;1)$ be the LOQ. The algorithm presented transforms any response value $x'$ towards the trait scale [0; 100] with due regard to *p*. In the following, if not otherwise stated, assume $L=0$, $U=100$, $r=\{1,2,\ldots ,k+1\}$ ($k\in {\mathbb {N}}$).

1. Choose $p\in (0;1)$. Set $lLOQ=100\cdot p/2$ and $uLOQ=100\cdot (1-p/2)$ (e.g., $p=0.05$, lLOQ=2.5, uLOQ=97.5)
2. Define the $range:=uLOQ-lLOQ$ and the step width $sw:=range/k$ (e.g., $range=;span class='convertEndash';97.5-2.5;/span;=95$ and $sw=95/4=23.75$).
3. Let the observed response value be $x'=r_s\in \{r_1,\ldots ,r_{k+1}\}$ with $s\in \{1,\ldots ,k+1\}$ (e.g., $x'=3$ corresponds to $s=3$).
4. Calculate the response value $x^*=lLOQ+sw\cdot (s-1)$ (e.g., $x'=3$ and $x^*=2.5+23.75\cdot (<span class='convertEndash'>3-1</span>)=50$).

For example, using $p=0.05$ the above algorithm transforms the RS $r=\{1,2,3,4,5\}$ towards the RS* $r^*=\{2.5,26.25,50,73.75,97.5\}$. Please note that the bounds of $r^*$ depend on *p*. Any $x^*\in (lLOQ; uLOQ)$ represents the OMA while $100-x^*$ represents the OMD.

**Fig. 2:**
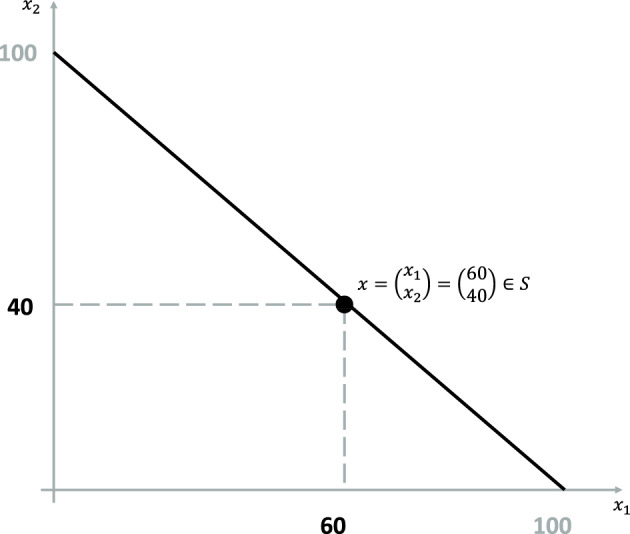
The Simplex of bipolar scales data is given by the black line. $x_1$ and $x_2$ represents the OMA and OMD towards the item assertion, respectively. The exemplary point $x=(60,40)^T$ illustrates and OMA of 60 and an OMD of 40. Figure according to [11]

Define $x=(x_1,x_2)^T\in {\mathbb {R}}^2$ with $x_1:=x^*$, $x_2:=100-x^*$, $x_1,x_2>0$ and $x_1+x_2=100$ where $()^T$ denotes the transpose. Generally, the compositional data space (the Simplex) is defined as ${\mathcal {S}}:=\{x=(x_1,\ldots ,x_D)^T\in {\mathbb {R}}^D\ |\ \sum _{i=1}^D{x_i}=\kappa \in {\mathbb {R}},\ x_i>0\ \forall \ i=1,\ldots ,D\}$. With $D=2$ and $\kappa =100$ *x* fulfills the definition of compositional data [4, 24–27]. An illustration of the Simplex of bipolar scales data is presented in Fig. 2

### Ilr and inverse ilr transformation

Any compositional data point *x* depends on the Aitchison metric [6]. However, most standard statistical procedures (e.g., computation of arithmetic means, Pearson correlation, (multiple) linear regression, t-tests) are based on the Euclidean metric. The ilr transformation yields interval scaled data underlying the Euclidean metric [28]. By means of the ilr and the inverse ilr, data and statistical results (e.g., mean values) can easily be (back-)transformed. The ilr transformation is defined as $ilr(x)=ilr((x_1,\ldots ,x_D)^T):=(z_1,\ldots ,z_{D-1})^T$ with

1 $$\begin{aligned} z_s=\sqrt{\frac{s}{s+1}}\ln \frac{\root s \of {\prod _{j=1}^s{x_j}}}{x_{s+1}},\ s=1,\ldots ,D-1 \end{aligned}$$

In the present case of $D=2$ the ilr reduces to $ilr((x^*,100-x^*)^T)=z_1$ with

2 $$\begin{aligned} z_1=\sqrt{0.5}\ln \frac{x^*}{100-x^*}. \end{aligned}$$

For example, the ilr transform of the RS $r^*=\{2.5,26.25,50,73.75,97.5\}$ denotes $ilr((2.5,97.5)^T)=-2.59$, $ilr((26.25,73.75)^T)=-0.73$, $ilr((50,50)^T)=0$, $ilr((73.75,26.25)^T)=0.73$ and $ilr((97.5,2.5)^T)=2.59$.

Please note that the bounds of the ilr RS depend on *p* because the bounds of $r^*$ depend on *p*. The smaller $p\in (0,1)$ is, the closer are the bounds of $r^*$ to 0 and 100, respectively. Therefore, $\lim \limits _{p\rightarrow 0}\frac{r_1^*}{r_{k+1}^*}=0$, $\lim \limits _{p\rightarrow 0}\frac{r_{k+1}^*}{r_1^*}=\infty$ and $\lim \limits _{p\rightarrow 0}\ln {\frac{r_1^*}{r_{k+1}^*}}=-\infty$, $\lim \limits _{p\rightarrow 0}\ln {\frac{r_{k+1}^*}{r_1^*}}=\infty$, i.e., the spread of the ilr RS increases as $p\rightarrow 0$.

The inverse ilr is used to back-transform any $z\in {\mathbb {R}}^{D-1}$ to an $x\in {\mathcal {S}}$ yielding the Simplex representation of the data. The inverse ilr is defined as follows. Let $z=(z_1,\ldots ,z_{D-1})^T\in {\mathbb {R}}^{D-1}$.

3 $$\begin{aligned} y_s&:=\sum _{j=s}^D{\frac{z_j}{\sqrt{j(j+1)}}}-\sqrt{\frac{s-1}{s}}z_{s-1};\ z_0:=z_D:=0 \end{aligned}$$

4 $$\begin{aligned} x_s&:=\kappa \cdot \frac{e^{y_s}}{e^{y_1}+\ldots +e^{y_D}},\ s=1\ldots ,D \end{aligned}$$

Like the ilr, the inverse ilr is simplified in the present case. The corresponding $x^*$ is obtained by setting $z_0:=z_D:=0$ and $\kappa =100$ with

5 $$\begin{aligned} x^*=100\cdot \frac{e^{y_1}}{e^{y_1}+e^{y_2}}\ \text {with}\ y_1=\sqrt{0.5}z_1\ \text {and}\ y_2=-\sqrt{0.5}z_1. \end{aligned}$$

Again, the complete compositional data point is given by $x=(x^*,100-x^*)^T$. Applying the inverse ilr transformation to the ilr RS yields the RS $r^*$, e.g., invilr(0.73)=73.75 in the above example.

Please note that in the present case of $D=2$ the ilr transformation is nearly identical with the additive log-ratio (alr), the centered log-ratio (clr) and the logit transformation. Lehmann and Vogt [13] provide a deeper introduction to the different types of data transformation and discuss advantages and disadvantages.

The idea of data evaluation is straight forward:

1. Apply the ilr transformation to obtain interval-scaled data.
2. Analyse the ilr-transformed data using any appropriate statistical procedure (e.g., t-test, linear regression etc.)
3. Interpret the results on the interval scale.
4. If necessary: use the inverse ilr transformation to back-transform the results to the Simplex (e.g., apply the invilr to the arithmetic mean of ilr transformed data) and interpret.

### The simulation

Concerning two-sample t-tests it is common practice to use Cohen’s *d* (UNPAIRED) or $d_z$ (PAIRED) as a measure of effect size [29, 30]. It is well-known that many statistical procedures are robust against violations of the assumption of normally distributed data (e.g. the UNPAIRED and the PAIRED, see [18, 31]). That is, using the PAIRED and UNPAIRED with Laplace distributed data seems possible although the strict requirements of the t-tests are not fulfilled.

It is common practice to calculate means or sums of item responses when measuring personality traits. The CLT in its various versions (among them allowing for non i.i.d. random variables and other generalizations, see [32]) grants that means and sums are asymptotically normally distributed as long as the variances of the corresponding random variables are sufficiently small ([33]). As means and sums differ by the constant “1/*numberofobservations*” they are equivalent. Thus, the simulation focuses on means.

Imagine two groups (say, *a* and *b*) of individuals and a hypothetical personality trait *T*, (e.g., *T*=openness). Let $\zeta _i^a$ denote the order of magnitude of *T* of individual *i* of group *a* in the ilr-transformed space. Analogously, $\omega _i^b$ denotes the order of magnitude of *T* of individual *i* of group *b* in the ilr-transformed space. Concerning the PAIRED $\zeta _i^a$ and $\omega _i^b$ refer to the same individual. Whereas, concerning the UNPAIRED $\zeta _i^a$ and $\omega _i^b$ refer to different individuals.

Let $z_i^a$ and $w_i^b$ be the (simulated) means of the ilr-transformed item responses, that is, $z_i^a$ estimates $\zeta _i^a$ and $w_i^b$ estimates $\omega _i^b$. The simulation generates values $z_i^a$ and $w_i^b$ assuming different univariate distributions of two real valued random variables $Z^a,W^b$ with expectations $\mu _a,\mu _b\in {\mathbb {R}}$ and variances $\sigma _a^2,\sigma _b^2\in {\mathbb {R}}_{>0}$.

**Fig. 3:**
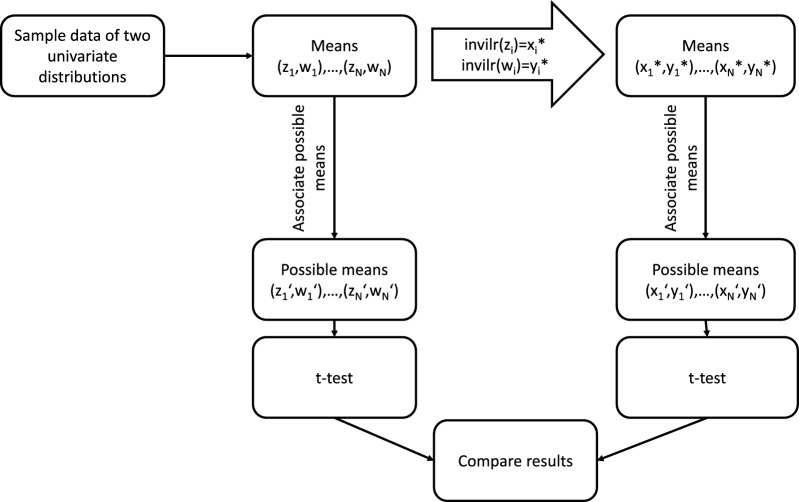
Simulated values are associated with their closest possible means (left-hand path). By means of the inverse ilr transformation the simulated values are transformed to the RS* and associated with their closest possible means on the original RS (right-hand path). $H_0:\ \mu _a-\mu _b=0$ is tested in both paths and the proportions of rejections of $H_0$ are obtained. Figure according to [11]

The univariate Laplace distribution is applied using the rlaplace() function of the R package VGAM (version 1.1–11). For details refer to [34]. An illustration of the procedure is presented in Fig. 3.

Calculating means of a finite number of item responses yields a discrete set of possible means. For example, using $K=k+1=5$, $p=0.05$ and $I=2$ items the ilr RS equals $\{-2.59,-0.73,0,0.73,2.59\}$. The corresponding set of possible means is given by $\{-2.59,-1.66,-1.30,-0.93,-0.73,-0.37,0,$ 0.37, 0.73, 0.93, $1.30,1.66,2.59\}$. Replacing any simulated mean $z_i^a$ $(i=1,\ldots ,N)$ with its nearest possible mean $\mu ^{ilr}_i$ according to the Euclidean metric yields realistic values. Let $z^a=0.82$ be a simulated mean. In the above example $\mu ^{ilr}=0.93$ denotes the nearest possible mean of $z^a=0.82$. Note that the number of possible means depends on the number of responses $K=k+1$ and the number of items $I\in {\mathbb {N}}$. Therefore, the simulation uses different numbers of items $I\in \{1,\ldots ,8\}$ and numbers of responses $k+1\in \{4,5,6\}$.

The inverse ilr transforms the simulated means to the RS* where they are replaced with their nearest possible mean, each. Although the Aitchison metric should be used on the RS*, the Euclidean metric is used to obtain the nearest possible means. This approach is necessary because in common practice means are calculated without considering the compositional structure of the response data. The intention of the simulation is to show the effects of disregarding the compositional structure during statistical analysis. Note that each mean of a set of item responses located on the RS* corresponds to a mean of the same set of item responses located on the original RS. Thus, any simulated mean $z_i^a$ $(i=1,\ldots ,N)$ can also be assigned to its nearest possible mean $\mu ^{orig}_i$ according to the original RS. For example, let $z^a=0.82$ be a simulated mean, then, invilr(0.82)=76.13. Consider the RS $r=\{1,2,3,4,5\}$ and the RS* with $r^*=\{2.5,26.25,50,73.75,97.5\}$ and a number of $I=2$ items. The sets of possible means (SPM) on both scales are $SPM^{RS}=\{1,1.5,2,2.5,3,3.5,4,4.5,5\}$ and $SPM^{RS^*}\{2.5,14.38,26.25,38.13,50,61.88,73.75,85.63,97.5\}$, respectively. The value 73.75 denotes the nearest possible mean of 76.13 on the RS* representing the mean $\mu ^{orig}=4$ on the initial RS.

Each simulation run generates two data sets: $ILR=\{(\mu ^{ilr}_{a,i},\mu ^{ilr}_{b,i})\ |\ i=1,\ldots ,N\}$ and $ORIG=\{(\mu ^{orig}_{a,i},\mu ^{orig}_{b,i})\ |\ i=1,\ldots ,N\}$. Consider the null hypothesis $H_0:\ \mu _a-\mu _b=0$. The t-test based on Student’s t-distribution is applied to both the ILR and ORIG data sets. The two proportions of rejections of $H_0$ in 1000 runs represent the estimated statistical powers of the t-test on both scales, that is, $Power^{ilr}$ and $Power^{orig}$. The difference $\Delta Power=Power^{ilr}-Power^{orig}$ indicates the superiority or inferiority of the ilr approach. Welch’s modification of the UNPAIRED is used if and only if the variances of the underlying DGPs are unequal.

### Parameters of the simulation study on two-sample t-tests

The simulations of the PAIRED (UNPAIRED) consider 3,600 (3,600) scenarios each consisting of 1,000 simulation runs. Lehmann and Vogt [4, 11, 13] revealed interesting ranges of specific parameters. For example, the number of Items *I* seems to be influential if $I\in \{1,2,4,6\}$. Concerning $I\ge 8$ results hardly vary. $\Delta Power$ seems to depend on the granularity of the response scale $K=k+1\{4,5,6\}$. Different sample sizes $N_1,N_2\in \{50,100\}$ should also be considered. Additionally, define $s^2=s_{a}^2+s_{b}^2$ as the total variance representing the overall dispersion. The underlying variances are $s_{a}^2\le s_{b}^2\in \{0.08,0.32,0.72,1.28\}$. Additionally, the LOQ $p\in \{0.05,0.1,0.2\}$ can affect the results.

Please note, that for a Laplace distribution with probability density function

6 $$\begin{aligned} f(x)=\frac{1}{2b}e^{-\frac{\left| x-\mu \right| }{b}} \end{aligned}$$

the mean equals $\mu$ and variance equals $2b^2$. Choosing $b\in \{0.2,0.4,0.6,0.8\}$ yields the above variances $s^2_a$ and $s^2_b$.

We choose the simulation parameters similar to [4, 11, 13, 35–37]. This offers several advantages, which will be examined in more detail below.

1. The choice of identical parameters supports the comparability of results with the aforementioned studies.
2. With an increasing number of items *I*, the CLT postulates a normal approximation of item means and sum scores. Since this is to be explicitly excluded here, it is necessary to allow only small numbers of items. Furthermore, the studies mentioned above suggest that hardly any differences are to be expected for $I\ge 8$.
3. The granularity of the response scale $\{1,\ldots ,k+1\}$ could influence $\Delta Power$, as it, together with the number of items *I*, determines the range of possible item means. It is therefore necessary to vary $k+1$. Numerous established practical scales use the response format $\{1,\ldots ,k+1\}$ with $k+1 \in \{4,5,6\}$ [38].
4. The LOQ is both a mathematical and a psychometric parameter. A *p* close to 0 (e.g., 0.05) means that the considered Likert scale covers a broad range (e.g., 95%) of the construct scale, i.e., the Likert scale is of high quality. Using a high-quality scale is desirable from a psychometric perspective. However, the quality of the scale depends not only on the formulation of the items but also on the complexity of the construct under consideration. The more complex a construct is, the more facets it has. These must be represented using corresponding items. The better an item’s formulation captures the associated facet in terms of content, the lower the value of *p*. In fact, there are qualitative differences in the various formulation possibilities of an item to capture a facet of the construct, leading to different LOQs. An illustrative case in this context is the evaluation of the Big 5 personality traits using different measurement instruments. While the BFI-10 includes 2 items per trait, the NEO-FFI offers 12 items per trait. Evaluating an individual’s personality with only two validated items cannot match the precision of a measurement carried out with 12 validated items. It is therefore necessary to vary the value of the LOQ. We choose the *p* so that it represents a scale of high quality ($p=0.05$), medium to high quality ($p=0.1$), and low to medium quality ($p=0.2$).
5. While [11, 12] chose steps of 0.05 between different Cohen’s *D* values ($D\in \{d,d_z\}$) their results suggest that larger steps of width 0.2 do not cause a serious loss of information or quality. Thus, we choose $D\in \{0.2,0.4,0.6,0.8,1\}$.
6. Societies may exhibit varying degrees of liberalism and open-mindedness, leading to a range of expressions of traits within different populations. For instance, the levels of openness or diversity competence can differ significantly between intolerant and liberal societies, resulting in a wider or narrower variance in the manifestation of these traits. Additionally, the variance is influenced by the specific population under consideration, whether it be a single country, a union of countries, or an entire continent. Consequently, the potential range of values for a construct can vary, indicating a greater or lesser variance in the DGP. Practically, means and variances of the DGP should be chosen such that the simulated data are likely to fall into to interval of possible manifestations. For example, setting $$K=k+1=5$$ and $$p=0.05$$ the ilr RS and the corresponding set of possible means is limited to the interval (−2.59,2.59). Therefore, the means $$\mu _a=\frac{D\sqrt{(s_{a}^2+s_{b}^2)/2}}{2}$$ and $$\mu _b=-\frac{D\sqrt{(s_{a}^2+s_{b}^2)/2}}{2}$$ are located symmetrically around 0 and the variances $$s_a^2$$ and $$s_b^2$$ are small. The total variation $$s^2$$ affects the effect size in the form of Cohen’s *d* and $$d_z$$ [29]. Given a fixed effect size, increasing the total variation leads to an increase in the distance between population means. These move away from each other towards the limits of the support of possible item means. This, in turn, affects how likely it is to observe a test person with an extremely high or low expression of the construct. Using the example of an ilr transformed response scale $$\{-2.59,-0.73,0,0.73,2.59\}$$ ($$p=0.05$$ with $$k+1=5$$), one can see that the absolute range is 2*2.59=5.18. According to Eq. (6) the variance is given by 2b² and the standard deviation by $$\sqrt{2}b$$. The simulated item means are replaced by the nearest possible item mean. Considering the limited support of possible item means, care must be taken to ensure that the simulation generates values outside the range as rarely as possible, as these would always be replaced by the maximum or minimum possible item mean. This would favor a concentration of simulated values at the edges of the support and distort the results. We have therefore decided to vary the parameter *b* in equidistant small steps of $$\{0.2, 0.4, 0.6, 0.8\}$$. The resulting maximum standard deviation is thus $$\sqrt{1.28}=1.13$$. If one expects that mostly values in the range of $$\pm h$$ standard deviations around the population mean are generated, the range of expected values around the population mean $$\mu$$ would be approximately $$\mu \pm 2*h*1.13$$ ($$\mu \in \{\mu _a,\mu _b\}$$). For h=1 (1.5), one would thus obtain a range of 2.26 (3.39), which, depending on the chosen value for *p*, corresponds to about half the range of the support. Considering the fact that the item means of two Likert scales are simulated, it can be assumed that both corresponding ranges approximately cover the set of all possible item means.
7. It is widely recognized that the number of responses on a scale (e.g., ${1,\ldots ,k+1}$ where $k+1=K\in {\mathbb {N}}$) does not impact the validity of a psychometric scale ([39]), but increasing *K* can improve the reliability of measurements ([40]). Research by [12–14]) suggests that the number of responses *K* on a scale can influence the outcomes of statistical analyses. The number of test persons *N* affects statistical power. It is expected that with an increasing number of observations, statistical power increases at a constant effect size [29]. In practice, the number of test persons varies greatly. In the context of representative surveys, sample sizes of more than 1000 persons are sometimes sought, whereas other studies may have fewer than 100 test persons due to a limited survey period. From a theoretical perspective, it is expected that an increase in the number of persons leads to a left shift of the power curve. A too large *N* could result in the statistical power of PAIRED and UNPAIRED being almost 100% across all parameter constellations, regardless of whether the ilr transformation is applied. This would make it potentially impossible to detect effects. We have therefore decided on small numbers of persons $N \in \{50, 100\}$.

### Results of the simulation

**Fig. 4:**
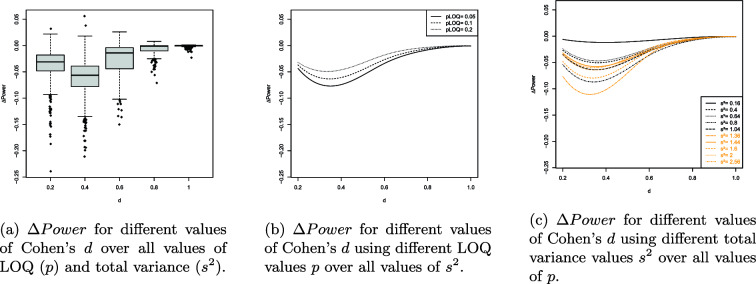
$\Delta Power$ values of the UNPAIRED

**Fig. 5:**
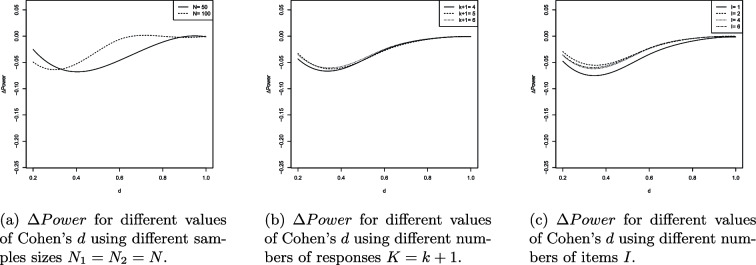
Splines of $\Delta Power$ values of the UNPAIRED

**Fig. 6:**
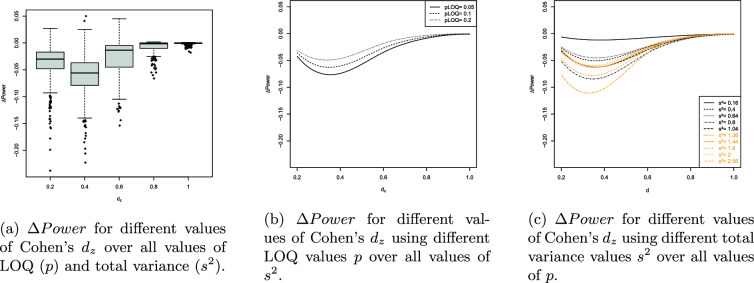
$\Delta Power$ values of the PAIRED

**Fig. 7:**
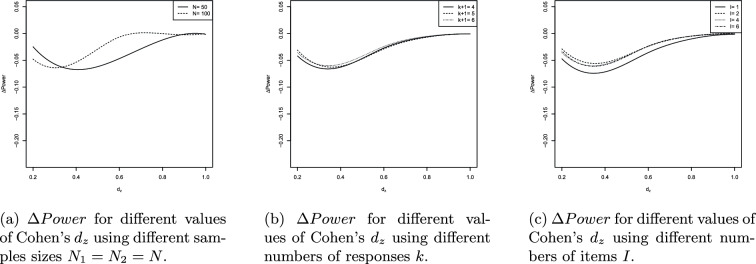
Splines of $\Delta Power$ values of the PAIRED

The results of the UNPAIRED and PAIRED are visualized in Figures 4a-5c and 6a-7c. The splinefun function of the R statistic software package is used to create the splines. For details refer to the fmm method of [41].

The results can be summarized as follows:

1. On average, the ilr approach causes a loss of statistical power because $\Delta Power<0$ in most scenarios evaluated.
2. $\Delta Power$ is most negative if $d=0.4=d_z$.
3. As *p* increases $\left| \Delta Power\right|$ decreases.
4. $\left| \Delta Power\right|$ is near 0 if the total variance $s^2$ is near 0. Increasing the total variance increases $\left| \Delta Power\right|$.
5. As the sample sizes $N_1$ and $N_2$ increase the extremum of the $\Delta Power$ curve is displaced towards decreasing values of *d* and $d_z$. For example, consider the PAIRED with $N_1=N_2=50$. The extremum of the $\Delta Power$ curve is located at $d=0.4$ and $\Delta Power$ is close to 0 if $d\ge 0.9$. Regarding $N_1=N_2=100$ the extremum of the $\Delta Power$ curve is located at $d=0.3$ and $\Delta Power$ is close to 0 if $d\ge 0.6$.
6. The number of responses hardly affects $\Delta Power$.
7. If $I>1$ the number of items does not affect $\Delta Power$. However, $I=1$ increases $\left| \Delta Power\right|$.

## Discussion and limitations

Lehmann and Vogt [4, 11, 13] showed an increase of statistical power caused by the ilr approach. Seen from this angle, the results of the current simulations seem surprising.

However, the ilr-induced loss of statistical power is plausible. First, consider some general effects. Heavy-tailed distributions provide more extreme observations with larger probability than, e.g., a normal distribution does (assuming identical means and variances). As a consequence, the associated possible means will more likely be located at the edge areas of a response scales. That is, the dispersion of the data increases reducing the statistical power of the PAIRED and UNPAIRED. The spread of the ilr response scale depends on the value of *p* representing the LOQ. For example, consider the original RS $RS^{orig}=\{1,2,3,4,5\}$. Applying $p=0.02$ yields the ilr transformed RS $RS^{ilr}_{p=0.02}=\{-3.25, -0.76, 0, 0.76, 3.25\}$, whereas, $p=0.2$ yields $RS^{ilr}_{p=0.2}=\{-1.55, -0.60, 0, 0.60, 1.55\}$. Small values of *p* close to 0 cause a large spread of the ilr RS.

Contrary to the original RS the ilr RS does not provide equidistant responses. Compare the relative distances of the edge responses 5 and 4, 3.25 and 0.76, 1.55 and 0.60. We have $(5-4)/(5-1)=0.25$, $(3.25-0.76)/(3.26-(-3.26))=0.38$ and $(1.55-0.66)/(1.55-(-1.55))=0.29$. Random values of a heavy-tailed DGP will more likely be located at the edge areas of the support and their relative distances will be larger on the ilr RS than on the original RS. Consequently, the dispersion on the ilr RS is larger reducing the statistical power of the PAIRED and UNPAIRED. Additionally, the relative distance of the edge responses increases as $p\rightarrow 0$ enhancing the loss of statistical power. The arguments are supported by Fig. 4b and 6b showing that decreasing $p\rightarrow 0$ increases the loss of statistical power.

The reduction in statistical power can also be attributed to a clustering effect at the ends of the response scale. As can be seen, the loss of statistical power is highest for large values of the total variation $s^2$ (cf. Figure 4c). The total variation is maximal when both variances $s_a^2$ and $s_b^2$ are maximal, i.e., $s_a^2=s_b^2=1.28$ and $s^2=s_a^2+s_b^2=2.56$. Large variance implies that the simulated values are likely to deviate strongly in both directions from the corresponding population mean. Since the values are replaced by the nearest possible item means, the simulation thus provides a large number of values for both constructs that coincide with the boundary values of the support. This applies to both the ilr transformed and untransformed item means. For $p\in \{0.05, 0.1\}$, the ilr transformation broadens the range of values, causing the clustering effect in the space of ilr transformed data to lead to greater dispersion than in the original data space (e.g., the interval [1; 5]). Consequently, it is expected that the effect size Cohen’s d in the ilr transformed data space is smaller than in the untransformed space. This effect is most pronounced when the total variation is maximal (cf. Figure 4c). The smaller the effect size, the lower the statistical power (ceteris paribus). The statistical power must be lower because the data tend to be more dispersed in the ilr transformed data space than on the original response scale. As *p* increases (e.g., $p=0.2$), the range of the ilr response scale and thus the support decreases. Since in the ilr transformed data space, the possible item means are not equidistant and the relative distances are particularly larger at the edges than in the original data space, the clustering effect has a stronger impact in the ilr transformed space even when *p* is increased. Although an increase in *p* leads to a reduction in the impact of the clustering effect on $\Delta Power$, it cannot completely eliminate the clustering effect (cf. Figure 4b).

Let’s examine the continuous univariate Laplace distribution characterized by a center at 0 and a small variance. This distribution is unimodal with a peak, exhibiting probability mass at its outer regions and displaying lower kurtosis compared to a normal distribution. The heavy tails of a Laplace DGP make it more prone to generating large absolute values in contrast to a normally distributed DGP. Conversely, the small variance ensures that values near 0 are highly probable. That is, the heavy tails are “less heavy” if the variance is small. Thus, sampled values will be close to the center and large values are less probable. As a result, *d* and $d_z$ increase as well as the statistical power of the t-test applied to ilr transformed data (compare the spline corresponding to small values of $s^2$ in Fig. 4c and 6c). If $s^2$ is near 0 the ilr approach hardly affects the statistical power of the PAIRED and UNPAIRED.

When *I* is greater than or equal to 2, the number of items does not seem to have a significant impact on $\Delta \ Power$ (compare Fig. 5c and 7c). The set *PM* of possible means is influenced by *I*; when *I* is at least 2, *PM* appears to be sufficiently large and dense, reducing the heavy-tail effect that leads to extreme values. In other words, increasing *I* results in an increase in the number of possible means in the edge areas, causing the heavy-tailed DGP to produce values other than just the extremes. In cases where *I* equals 1, *PM* aligns with the RS. The *RD* between adjacent responses increases when responses are situated in edge areas, leading to more extreme possible means compared to scenarios with *I* greater than or equal to 2 items.

The boxplots of Fig. 4a and 6a provide additional information. The trend of the medians is very similar to the trend of the splines. In the range $0.2\le \left| D\right| \le 0.6$ ($D\in \{d,d_z\}$), the heights of the boxes and the lengths of the whiskers are largest. Outside that range the box heights and whisker lengths reduce. That is, the results are similarly reliable in that range. The height of the boxes is approximately 2 to 3 percentage points, indicating that the boxes represent a range of median ± 1% or ± 1.5%. Taken together, both pieces of information suggest a qualitatively adequate robustness of the results.

The values outside the whiskers indicate that there are scenarios that cause even more extreme changes in statistical power. Generally, there are less values above the upper whisker than below the lower whisker. This means that when extreme deviations occur, they tend to indicate an additional decrease in statistical power induced by the ilr approach. Qualitatively, the range of extreme power loss is greater than the range of minor power gain.

However, if $\{d,d_z\}\ni D$ outside (0.2, 0.6) the boxes narrow and the whiskers shorten, indicating an increasing robustness of the results. As $D\rightarrow 0$ or $D\rightarrow 1$ we have $\Delta \ Power\rightarrow 0$ and the extreme deviations distribute equally around the (more narrow) boxes. This is because the statistical power of the PAIRED and UNPAIRED increases (decreases) with increasing (decreasing) effect size. This increase (decrease) occurs regardless of whether the data are analyzed traditionally or using the ilr approach. In both cases, the statistical power converges to 1 (0) and therefore the difference converges to 0.

Overvall, the result can be considered robust because the pattern of the medians of the boxplots is similar to the splines and the widest boxes cover a short range of median±1%. The loss in statistical power using the ilr approach is evident if the DGP is heavy-tailed.

Exemplary, the real data re-analyses of [4, 11] and [42] showed that more significant results can be expected when using the ilr approach with the PAIRED and UNPAIRED supporting the simulations using normally distributed DGP. That is, the results of the real data re-analyses are contradictory to the simulation outcomes of the present study.

Although the violation of the CLT assuming a Laplace distributed DGP is questionable the simulation scenarios considered are important because they consider possible extremes and the parameters investigated are realistic. Often, psychometric scales consist of a sufficiently large number of items and means or sums of ilr transformed item responses can be considered approximately normally distributed. On the other hand, there exist scales consisting of few items, e.g. the BFI-10 scale (2 items per Big-5 personality trait [43]) and the RS-1 scale (1 item measuring risk disposition [44]).

Finite response scales and finite numbers of items imply finite item response means and sums. Consequently, the variance of the distribution of means or sums cannot approach infinity. Reconsider the ilr transformation and the parameter *p*. Let $x_1=(p/2, 100-p/2)^T,x_2=(100-p/2, p/2)^T\in {\mathcal {S}}$, then $ilr(x_1)\rightarrow -\infty$ and $ilr(x_2)\rightarrow \infty$ as $p\rightarrow 0$ yielding an approximately infinite support of the item responses and all possible means and sums. Therefore, the distribution of means and sums can only have approximately infinite variance. To that effect, only a heavy-tailed distribution of finite variance (e.g., the Laplace distribution) is reasonable.

Furthermore, $p\approx 0$ does not seem realistic. The simulation considered values $p\in \{0.05,0.1,0.2\}$ resulting in ilr transformed response scales ranging from (−2.59, 2.59) (p=0.05) to (−1.55, 1.55) (p=0.2). That is, $p=0.05$ yields a support of possible ilr transformed response values ranging from −2.59 to 2.59 rendering the assumption of an approximately heavy-tailed Laplace distribution of means less realistic.

Altogether, Laplace could be a possible DGP if the CLT is violated and $p\approx 0$, that is, when considering scales with a small number of items and *p* very close to 0. On the other hand, the smaller the number of items of a scale, the larger the LOQ, that is, the larger *p*. Consequently, $p\approx 0$ can hardly occur if the number of items is small. In this sense, the Laplace distribution can be considered as an extreme DGP of reduced practical relevance.

Differences in means form a basic instrument in the analysis of big data sets in psychological healthcare possibly consisting of large numbers of groups and subgroups of patients. The PAIRED and UNPAIRED as well as the analysis of variance (ANOVA) are special cases of the linear regression model assuming normally distributed residuals. Thus, the results obtained generalize towards a much larger set of statistical procedures. For details refer to [33]. The loss in statistical power using the ilr approach is evident if the DGP is heavy-tailed.

In practical applications, it is first necessary to clarify whether a correlational relationship between sychometric variables (e.g., constructs) or a grouping effect (e.g., expression of a construct divided by gender) should be investigated. The results of [4, 13, 36, 37] show that an increase in statistical power can generally be expected in the context of correlational relationships. However, an increase in power for grouped data has only been demonstrated so far if the item means are approximately normally distributed [11, 12, 42]. The prediction by the central limit theorem must therefore hold to expect an increase in statistical power. Lehmann and Vogt [14, 15] showed that the ilr approach increases the normal approximation of item means. However, whether this is sufficiently given must be tested. It is therefore recommended to perform a test for deviations from the normal distribution (e.g., the Shapiro-Wilk test [45], the Shapiro-Francia test [46], the Anderson-Darling test [47]) and to consider a histogram and/or density plot of the data before applying t-tests or analyses of variance. However, if the null hypothesis of a test against normality is rejected and/or the plots indicate a non-normal distribution this does not imply that the data come from a heavy-tailed distribution. Please note that the Anderson-Darling test is theoretically capable of testing whether there is a deviation from any assumed underlying distribution. Critical values, however, exist only for testing against the normal distribution, which limits its application with respect to the class of heavy-tailed distributions. From a practical point of view, there are therefore two ways: (1) analysis of the data without ilr transformation and (2) analysis of the data with ilr transformation. Ultimately, statistics only provide indications of possible relationships or differences. Statistics cannot replace the examination of results for plausibility and content interpretation, so no general recommendation can be made. In the case of non-normal distribution, the data should therefore be analyzed using both method (1) and method (2), and the results should be examined for plausibility in terms of content. Neither method is generally preferable.

Overall, the DGPs considered are extremes rarely occurring in practice. Future research should focus on one-item scales with $p\not \approx 0$ using a non-heavy-tailed DGP. Furthermore, scales of $I\ge 2$ items should be considered assuming an approximately normally distributed DGP of means or sums of ilr transformed item responses. Also, the effects of skewed, bimodal and U-shaped DGPs should be considered.

## Data Availability

There are no real data available. The authors agree to share the R codes used in the simulation process upon request.

## References

[1] Tariq MU, Babar M, Poulin M, Khattak AS, Alshehri MD, Kaleem S (2021) Human behavior analysis using intelligent big data analytics. Front Psychol 12:686610 34295289 10.3389/fpsyg.2021.686610 PMC8290162

[2] Cheung MW-L, Jak S (2016) Analyzing big data in psychology: a split/analyze/meta-analyze approach. Front Psychol 7:738 27242639 10.3389/fpsyg.2016.00738 PMC4876837

[3] Cheung MW-L, Jak S (2018) Challenges of big data analyses and applications in psychology. Zeitschrift für Psychologie 226(4):209–211

[4] Lehmann, R. and Vogt, B. (2023b). Reconsidering bipolar scales data as compositional data improves psychometric healthcare data analytics. In *Proceedings of the 56th Hawaii International Conference on System Sciences*, pages 2380–2389

[5] Aitchison J (1986) The statistical analysis of compositional data. Chapman and Hall

[6] Filzmoser P, Hron K, Reimann C (2009) Univariate statistical analysis of environmental (compositional) data: problems and possibilities. Sci Total Environ 407:6100–6108 19740525 10.1016/j.scitotenv.2009.08.008

[7] Aitchison, J. (2003b). *The statistical Analysis of Compositional Data*. Blackburn Press, reprint of 1986 containing additional material edition

[8] Filzmoser P, Hron K (2008) Outlier detection for compositional data using robust methods. Mathematical Geosci 40:233–248

[9] Lehmann R (2014) A new approach for assessing the state of environment using isometric log-ratio transformation and outlier detection for computation of mean pcdd/f patterns in biota. Environ Monitor Assess 187(1):4149 10.1007/s10661-014-4149-z 25427827

[10] Filzmoser P, Hron K (2009) Correlation analysis for compositional data. Mathematical Geosci 41:905–919

[11] Lehmann R, Vogt B (2023) Increasing the power of two-sample t-tests in health psychology using a compositional data approach. Cham, Springer Nature Switzerland

[12] Lehmann, R. and Vogt, B. (2024a). Compositional data statistics improves smart tourism data analytics: profound managerial decisions through reduced statistical bias and increased power. In *Proceedings of the 57th Hawaii International Conference on System Sciences*

[13] Lehmann R, Vogt B (2024) Improving likert scale big data analysis in psychometric health economics: reliability of the new compositional data approach. Brain Infor 11(1):19 10.1186/s40708-024-00232-z PMC11236837 38987395

[14] Lehmann, R. and Vogt, B. (2024d). Increasing normal approximation in psychometric health care data analyses using a compositional data approach. In *Proceedings of the 57th Hawaii International Conference on System Sciences*

[15] Lehmann, R. and Vogt, B. (2024g). Shifting psychometric bipolar scales data towards the normal distribution. In *Proceedings of the 57th Hawaii International Conference on System Sciences*

[16] Murphy J, Vallières F, Bentall RP, Shevlin M, McBride O, Hartman TK, McKay R, Bennett K, Mason L, Gibson-Miller J, Levita L, Martinez AP, Stocks TVA, Karatzias T, Hyland P (2021) Psychological characteristics associated withcovid-19 vaccine hesitancy and resistancein ireland and the united kingdom. Nat Commun 12(29):19 33397962 10.1038/s41467-020-20226-9 PMC7782692

[17] Pennycook G, Epstein Z, Mosleh M, Arechar AA, Eckles D, Rand DG (2021) Shifting attention to accuracy can reduce misinformation online. Nature 592(7855):590–595 33731933 10.1038/s41586-021-03344-2

[18] Carifio J, Perla RJ (2007) Ten common misunderstandings, misconceptions, persistent myths and urban legends about likert scales and likert response formats and their antidotes. J Soc Sci 3:106–116

[19] Carifio J, Perla RJ (2008) Resolving the 50 year debate around using and misusing likert scales. Med Educ 42:1150–1152 19120943 10.1111/j.1365-2923.2008.03172.x

[20] Likert R (1932) A technique for the measurement of attitudes. Archiv Psychol 22(140):5–55

[21] James J, Wood G (1988) The effects of incomplete information on the formation of attitudes toward behavioral alternatives. J Person Soc Psychol 54(4):580–591

[22] Loke WH (1989) The effects of framing and incomplete information on judgments. J Econ Psychol 10(3):329–341

[23] Romano A, Mosso C, Merlone U (2016) The role of incomplete information and others’ choice in reducing traffic: a pilot study. Front Psychol 7:135 26903931 10.3389/fpsyg.2016.00135 PMC4749693

[24] Aitchison, J. (2003a). *A Concise Guide to Compositional Data Analysis*. Department of Statistics University of Glasgow

[25] Aitchison J, Egozcue JJ (2005) Compositional data analysis: where are we and where should we be heading? Mathema Geol 37:829–850

[26] Aitchison J, Mateu-Figueras G, Ng K (2003) Characterization of distributional forms for compositional data and associated distributional tests. Mathematical Geol 35:667–680

[27] Aitchison J, Mateu-Figueras G, Ng KW (2003) Characterization of distributional forms for compositional data and associated distributional tests. Mathematical Geol 35:667–680

[28] Filzmoser P, Garrett RG, Reimann C (2005) Multivariate outlier detection in exploration geochemistry. Compu Geosci 31:579–587

[29] Cohen J (2013) Statistical Power Analysis for the Behavioral Sciences. Routledge

[30] Lakens D (2013) Calculating and reporting effect sizes to facilitate cumulative science: a practical primer for t-tests and anovas. Front Psychol 4:863 24324449 10.3389/fpsyg.2013.00863 PMC3840331

[31] Norman G (2010) Likert scales, levels of measurementand the laws of statistics. Adv Health Sci Educ 15:625–632 10.1007/s10459-010-9222-y 20146096

[32] Fischer H (2011) A History of the Central Limit Theorem. Springer

[33] Davidson J (2001) Econometric Theory. Blackwell Publishing, Oxford

[34] Yee, T. (2006). Vgam: Vector generalized linear and additive models

[35] Lehmann, R., Bengart, P., and Vogt, B. (2025). Discovering careless response behavior in psychometric data. In *Proceedings of the 58th Hawaii International Conference on System Sciences*

[36] Lehmann R, Vogt B (2024e) Robustness of the compositional data approach in bipolar psychometric likert scales big bimodal data analysis. In: Proceedings of the 10th IEEE International Conference on Engineering and Emerging Technologies (ICEET 2024). *IEEE Xplore* (in press)

[37] Lehmann, R. and Vogt, B. (2024f). Robustness of the compositional data approach in bipolar psychometric likert scales big skewed data analysis. In *Proceedings of the 23rd IEEE/WIC International Conference on Web Intelligence and Intelligent Agent Technology (WI-IAT).* IEEE WIC

[38] Spector P (1992) Summated Rating Scale Construction. SAGE Publications Inc, London

[39] Weijters B, Baumgartner H (2012) Misresponse to reversed and negated items in surveys: a review. J Mark Res 49(5):737–747

[40] Preston CC, Colman AM (2000) Optimal number of response categories in rating scales: reliability, validity, discriminating power, and respondent preferences. Acta Psychol 104(1):1–15 10.1016/s0001-6918(99)00050-5 10769936

[41] Forsythe GE, Malcolm MA, Moler CB (1977) Computer Methods for Mathematical Computations. Wiley

[42] Lehmann R, Vogt B (2024) Empirical insights into the value of a novel compositional data approach for analyzing bipolar Likert scale data. Lecture Notes in Computer Science, vol. 15541, chapter 27, ISSN 978-981-96-3293-0, Springer Nature Switzerland (in press)

[43] Rammstedt, B., Kemper, C. J., Klein, M. C., Beierlein, C., and Kovaleva, A. (2014). Big five inventory (bfi-10). *Zusammenstellung sozialwissenschaftlicher Items und Skalen (ZIS)*

[44] Beierlein, C., Kovaleva, A., Kemper, C. J., and Rammstedt, B. (2015). Kurzskala zur erfassung der risikobereitschaft (r-1). *Zusammenstellung sozialwissenschaftlicher Items und Skalen (ZIS)*

[45] Shapiro SS, Wilk MB (1965) An analysis of variance test for normality (complete samples). Biometrika 52:591–611

[46] Royston P (1993) A pocket-calculator algorithm for the shapiro-francia test for non-normality: an application to medicine. Statis Med 12(2):181–184 10.1002/sim.4780120209 8446812

[47] Anderson TW, Darling DA (1954) A test of goodness-of-fit. J Am Statis Assoc 49:765–769

